# Integrative transcriptomic analysis identifies meningeal-hippocampal immune communication in Alzheimer’s disease

**DOI:** 10.1186/s12967-026-08414-5

**Published:** 2026-06-25

**Authors:** Congcong Yan, Zhoushuai Chen, Yi Yan, Miao Wang, Wenting Chen, Feifan Zhou, Qian Liu

**Affiliations:** 1School of Biomedical Engineering, Hainan University, No. 7, University Road, Yazhou District, Sanya, Hainan Province 572025 China

**Keywords:** Alzheimer’s disease, Meninges, Microglia, Ligand-receptor signaling

## Abstract

**Background:**

Alzheimer’s disease (AD) exhibits spatial heterogeneity, yet the mechanisms by which immune-active meninges communicate with vulnerable brain regions during disease progression remain unclear. This study aimed to investigate meninges-hippocampus crosstalk and map the communication patterns during AD pathology development.

**Methods:**

We employed 5xFAD transgenic and PS19 tau-pathology mouse models and integrated bulk RNA sequencing, proteomics, and spatial transcriptomics across time-course analyses to distinguish AD pathology from normal aging processes. Transcription factor activity inference was coupled with ligand-receptor dynamics analysis to examine regulatory modules in homeostatic and disease-associated microglia.

**Results:**

We identified structure-specific disease genes, including a robust 19-gene hippocampal signature that was reproducible across an independent 5xFAD dataset and PS19 tau-pathology dataset. Spatial analysis defined a disease progression axis that transferred to the independent spatial dataset, where meningeal spots were enriched in advanced disease progression layers and disease modules showed consistent gradients. Ligand-receptor and microglial-state LRaxis analyses further supported candidate communication axes associated with pathology gradients and state-specific regulatory modules in both homeostatic and disease-associated microglia.

**Conclusions:**

Our spatial framework supports the meninges as a candidate modulatory interface associated with hippocampal pathology in AD mouse models. These mouse-derived, computationally inferred ligand-receptor and regulatory modules should be considered hypothesis-generating candidates that require validation in human AD tissues and functional perturbation studies before translational relevance can be inferred.

**Supplementary Information:**

The online version contains supplementary material available at https://doi.org/10.1186/s12967-026-08414-5.

## Introduction

AD accounts for approximately 60–80% of dementia cases globally, affecting over 50 million people worldwide with projections reaching 152 million by 2050 [1, 2]. The amyloid cascade hypothesis has dominated AD research, positing that amyloid-β (Aβ) accumulation initiates a pathological cascade leading to tau hyperphosphorylation, neuroinflammation, and neuronal loss [3, 4]. While bulk transcriptomics has determined the molecular characteristics of AD by analyzing changes in gene expression related to disease states, the spatial organization of disease progression and inter-regional communication networks remain poorly understood.

The meninges, traditionally viewed as protective membranes surrounding the brain, have recently been recognized as immunologically active structures housing diverse immune cell populations [5, 6]. Meningeal immune cells communicate with brain parenchyma through cytokines, growth factors, and other signaling molecules. Meningeal lymphatics provide a drainage pathway for brain interstitial fluid and facilitate immune surveillance [6–8]. Emerging evidence suggests that meningeal inflammation may contribute to and brain parenchyma in AD has not been systematically characterized. The role of meninges-brain communication in AD pathogenesis remains largely unexplored. Given that the hippocampus is one of the key brain regions affected early in AD, defining hippocampus-meninges signaling may reveal hypothesis-generating communication axes. Spatial transcriptomics technologies now enable simultaneous measurement of gene expression and tissue architecture at near-cellular resolution, and identification of intercellular communication patterns and mapping of disease-associated changes across anatomical regions [9, 10]. Recent studies have applied spatial transcriptomics to AD brains, revealing regional heterogeneity in disease-associated gene expression, but systematic analysis of the meningeal-brain parenchyma cross-border communication network is still relatively insufficient [11].

Here, we present an integrated transcriptomic analysis of AD mouse models combining bulk RNA-seq, proteomics, and spatial transcriptomics to achieve three objectives: (1) identify genes specifically associated with AD pathology rather than normal aging; (2) characterize the molecular communication between hippocampus and meninges during disease progression; and (3) elucidate the transcription factor-ligand-receptor networks regulating microglial states in AD. Our findings suggest a novel hippocampus-meninges communication axis associated with specific ligand-receptor pairs and identify shared and state-specific regulatory networks in homeostatic (M0) and disease-associated microglia.

## Materials and methods

### Datasets

We collected six public datasets: (1)E-MTAB-16,006 (dataset1, bulk RNA-seq, hippocampus and meninges, 5xFAD and wild-type (WT) mice at 3-, 6-, 9-months, *n* = 48 samples); (2) GSE198226 (dataset2, bulk RNA-seq, hippocampus, 5xFAD and WT at 2-, 5-, 8-months, *n* = 18 samples); (3) GSE218728 (dataset3, bulk RNA-seq, hippocampus, PS19 tau-pathology and age-matched WT mice at 4-, 9-months, *n* = 21 samples); (4) PXD030348 (proteomics, hippocampus, AD and WT at 2-, 5-, 8-months, *n* = 36 samples); (5) GSE174321 (spatial transcriptomics, whole brain sections, 5xFAD and WT mice at 3- and 7-months, *n* = 4 sections); and (6) GSE218360 (spatial transcriptomics, APP/PS1 AD mice, *n* = 8 sections).

### Data preprocessing

For genes with multiple transcript isoforms, we retained the isoform with the highest mean expression across all samples. Low-expression genes were filtered using two criteria: (1) genes expressed in fewer than 75% of samples were removed; (2) among remaining genes, those not expressed in at least one sample per experimental group were removed.

Gene expression matrices were extracted and converted to Fragments Per Kilobase of transcript per Million mapped reads (FPKM) using exon lengths calculated from the Mus musculus GRCm39 GTF annotation. A TxDb object was created using makeTxDbFromGFF, and exon lengths were computed by reducing overlapping exons and summing widths for each gene. FPKM values were calculated as: FPKM = (counts / gene_length_kb) / (total_counts / 10^− 6^).

Proteins were filtered to remove: (1) reverse database hits, (2) contaminants, (3) proteins with fewer than 2 unique peptides, and (4) protein expressed in fewer than 50% of the samples in each experimental group. For proteins with multiple gene names, only the first gene symbol was retained. For mice with the technical replication, we averaged protein expression in each group. The label-free quantification (LFQ) intensity values were extracted and log2-transformed after adding a pseudocount of 1.

### Differential expression analysis

Differential expression analysis was performed using DESeq2 (v1.34.0) with default parameters in bulk RNA-seq [12]. The DESeq function performed estimation of size factors, dispersion estimation, and negative binomial GLM fitting. Genes were classified as differentially expressed if they met thresholds of |Log_2_FC| > 0.58 (corresponding to 1.5-fold change) and false discovery rate (FDR) < 0.05. For each brain structure (hippocampus and meninges), we performed four comparisons: AD groups (9 M vs. 3 M, 6 M vs. 3 M) and WT aging controls (9 M vs. 3 M, 6 M vs. 3 M). Differential expression analysis was performed using limma (v3.50.0) in proteomics [13].

### Identification of disease-specific genes

Disease-specific genes were identified using a three-step set operation approach: (1) For each brain structure, we identified genes differentially expressed in AD by taking the intersection of 9 M vs. 3 M DEGs and 6 M vs. 3 M DEGs, ensuring genes showed consistent directional changes across both time points. (2) Similarly, we identified genes differentially expressed during normal aging in WT mice using the same intersection approach. (3) Disease-specific genes were defined as the set difference between AD DEGs and WT aging DEGs (AD DEGs minus WT DEGs), isolating genes altered in disease but not in normal aging. Disease gene module scores were calculated using AddModuleScore function.

### Functional enrichment analysis

Functional enrichment analysis was performed using Metascape (http://metascape.org) with default parameters. Gene lists were submitted for Gene Ontology (GO) Biological Process, Molecular Function, and Cellular Component enrichment, as well as KEGG pathway analysis. Enrichment results were filtered at FDR < 0.05 and visualized using Cytoscape (v3.9.1).

### Spatial transcriptomics analysis

Spatial transcriptomics data (GSE174321) were processed using Seurat (v5.0.0) with the following workflow [14]. Spots were filtered to retain those with nFeature_Spatial > 200 and percent.mt < 20, removing low-quality spots and potential damaged cells. Each sample was independently normalized using SCTransform, which performs variance stabilization and removes technical variation while preserving biological heterogeneity. Variable features were selected using SelectIntegrationFeatures (nfeatures = 3000) across all samples. Principal component analysis (PCA) was performed using RunPCA, followed by graph-based clustering using FindNeighbors (dims = 1:30) and FindClusters (resolution = 0.3). UMAP dimensionality reduction was performed using RunUMAP (dims = 1:30). Cell type enrichment scores were calculated using AddModuleScore function. For GSE218360, spot-level expression profiles were projected onto the DPA reference layers derived from 7-month GSE174321 samples. Diseaes module scores were recalculated in the validation dataset.

### Disease progression axis (DPA)

The DPA was defined to stratify samples by disease severity. We first calculated immune and synapse scores using AddModuleScore with gene sets for immune response and synaptic function (Supplementary Table 1). The DPA was then computed as:

$$\:DPA=Z\left(Immun{e}_{score}\right)-Z(Synapse\_score)$$

where Z represents Min-Max normalization within each time point (3 M and 7 M). Higher DPA values indicate more advanced disease pathology (elevated immune activation, reduced synaptic function). Samples were stratified into four disease stages (L1-L4) based on DPA quartiles within each time point. This stratification approach controls for baseline differences between time points while capturing disease progression gradients within each time point.

### Ligand-receptor analysis

Ligand-receptor pairs (LR) were obtained from CellPhoneDB (v2.0), a curated database of ligand-receptor interactions [15]. For each spatial spot, we calculated ligand and receptor gene set enrichment scores using single-sample Gene Set Enrichment Analysis (ssGSEA) on the ligand and receptor gene lists separately. To identify directional communication patterns, we compared correlations separately for hippocampus (receptor-disease vs. ligand-disease) and meninges (ligand-disease vs. receptor-disease). We constructed a ligand-receptor axis (LRaxis) by multiplying meninges ligand scores with hippocampus receptor scores for each LR pair, identifying the top 30 pairs with strongest positive correlation to hippocampus disease modules.

### Transcription factor activity analysis

First, we take the intersection of the gene set from the mouse DoRothEA regulatory network and the expression matrix, retaining only the target gene set observable in the data [16]. To reduce memory consumption per computation, we process spots in batches with a chunk size of 200. For each batch, we convert the submatrix to a dense matrix and calculate the TF activity score using the univariate linear model (ULM) method. Subsequently, we retaining the top 250 TFs with the highest variance, a linear regression model was constructed to evaluate its association with the LR disease course axis for each retained TF:

$$\:{TF}_{i}\:\sim\:LRaxis\_pos+Layer$$

Layer was used to control for confounding effects of DPA stratification. The candidate TFs are sorted by significance to obtain the candidate TFs most relevant to the LR axis.

Furthermore, to distinguish between “disease-shared” and “disease-specific” TF-LR coupling, we performed conditional correlation analysis on the high abundance spots of M0 and DAM (> 70%). To control for potential confounding factors, we constructed a design matrix.

$$X\, = \, \sim \,Layer\, + \,sende{r_{meninges}}\, + \,M0\, + \,DAM$$

The TF activity matrix and the corresponding LR activity matrix were then residualized (ordinary least squares regression was performed on each feature and the residuals were taken, The Spearman correlation coefficient matrix (TF×LR) is then calculated in the residual space, and the significance of the correlation is approximated using a t-distribution.:

$$\:{Y}_{res}=(I-{X\left({X}^{T}X\right)}^{-1}{X}^{T})Y$$

$$\:t=r\sqrt{\frac{n-2}{1-{r}^{2}}}\:,\:P=2*\mathrm{P}\mathrm{r}\left({|T}_{n-2}\right|\:\ge\:\left|t\right|)$$

Where n is the number of spots in the subset, and r is the correlation coefficient of TF–LR after decontamination.

### Statistical analysis

All statistical analyses were performed in R (v4.2.0). Venn graph visualization is implemented using the R package “VennDetail”. For correlation analyses, we report Spearman correlation coefficients with 95% confidence intervals computed using R package “stats”. For stratified correlation analysis in spatial data, we used linear regression with interaction terms to test for differences in slopes between disease stages. All p-values reported are two-sided unless otherwise specified.

## Results

### Identification of disease-specific genes in AD hippocampus and meninges

**Fig. 1:**
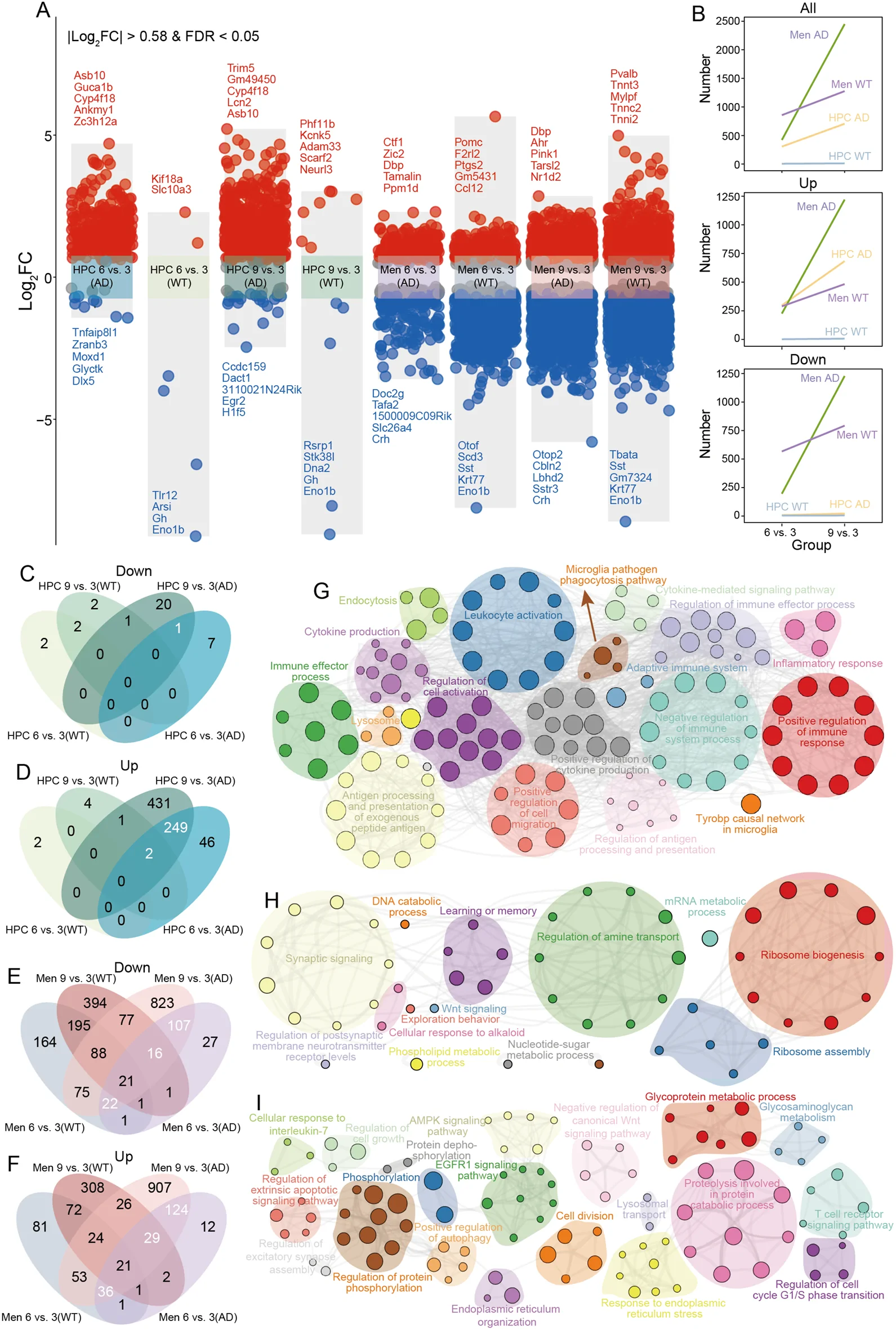
Disease-specific genes distinguish AD pathology from normal aging.** A** Volcano plots showing DEGs in hippocampus and meninges of AD transgenic mice compared to WT controls at 6 and 9 months versus 3 months baseline. Red dots represent significantly upregulated genes, blue dots represent significantly downregulated genes, and gray dots represent non-significant genes. **B** Line plots displaying the temporal progression of differentially expressed genes in AD compared to WT controls at 6 and 9 months versus 3 months. Separate lines show up-regulated (red) and down-regulated (blue) genes for hippocampus and meninges. **C-F** Venn diagrams illustrating the strategy for identifying disease-specific genes in (**C**, **D**) hippocampus and (**E**, **F**) meninges. Numbers in overlapping regions indicate shared genes between time points. **G-I** Gene Ontology (GO) and KEGG pathway enrichment analysis of (**G**) hippocampus-specific up-regulated genes (**H**) meninges-specific up-regulated genes (**I**) meninges-specific down-regulated genes using Metascape bioinformatics resources. FC: fold change

To identify transcriptional changes specific to AD pathology rather than normal aging, we performed bulk RNA-seq on hippocampal and meningeal tissues from 5xFAD transgenic mice and WT mice at 3-, 6-, and 9-months of age (Fig. 1A). After quality filtering, 14,965 genes were retained for downstream analysis. Differential expression analysis using DESeq2 with thresholds of |Log_2_FC| > 0.58 and FDR < 0.05 identified progressively more differentially expressed genes (DEGs) with disease progression in both brain structures (Fig. 1B). In the hippocampus, comparison of later time points (6 M and 9 M) versus the baseline (3 M) at AD and WT revealed 305 (AD) and 6 DEGs (WT) at 6 M, increasing to 705 (AD) and 12 DEGs (WT) at 9 M. The meninges exhibited more pronounced transcriptional changes, with 422 (AD) and 856 DEGs (WT) at 6 M, 2,449 (AD) and 1,276 DEGs (WT) at 9 M. Temporal analysis within genotypes showed that aging-associated changes in WT mice were substantially fewer than disease-associated changes in AD mice, suggesting that AD pathology accelerates transcriptional dysregulation beyond normal aging.

To distinguish disease-specific genes from aging-associated changes, we employed a comparative framework. For each brain structure, we defined: (1) aging-associated genes as those differentially expressed in both WT and AD aging comparisons (6 M vs. 3 M, 9 M vs. 3 M), and (2) disease-specific genes as those differentially expressed only in AD aging but not in WT aging. This analysis identified 251 disease-specific up-regulated genes in the hippocampus, with minimal down-regulated genes (1 gene; Fig. 1C, D). In the meninges, we identified 189 disease-specific up-regulated and 145 down-regulated genes (Fig. 1E, F). Notably, no genes overlapped between hippocampal and meningeal disease-specific up-regulated sets, indicating structure-specific transcriptional responses to AD pathology.

Functional enrichment analysis revealed distinct biological processes associated with disease specific genes in each brain structure (Fig. 1G). Hippocampus-specific upregulated genes were significantly enriched for immune response pathways and antigen presentation, consistent with the known role of neuroinflammation in AD pathogenesis [3]. Meninges-specific upregulated genes showed enrichment for cell signaling and immune cell interaction pathways, suggesting active intercellular communication at the brain-immune interface. Meninges-specific downregulated genes were associated with basic cellular activities and signal regulation, indicating potential impairment of normal meningeal function in AD.

### Validation of disease-specific genes in an independent cohort

**Fig. 2:**
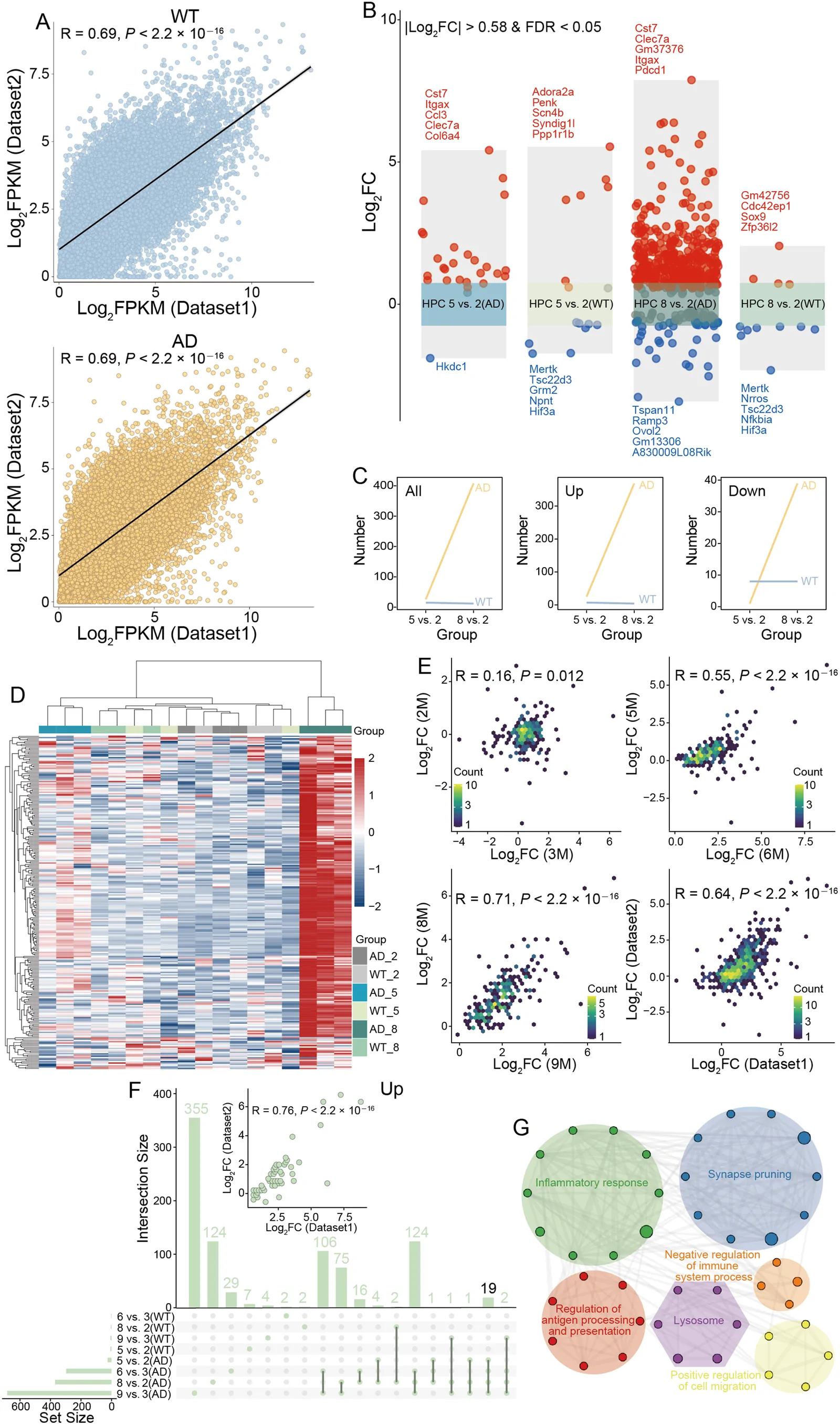
Independent cohort validates 19 robustly upregulated gene.** A** Scatter plot showing Spearman correlation of gene expression profiles between dataset1 and dataset2. Each dot represents one gene, with correlation coefficient (r) and p-value displayed. **B** Volcano plots showing DEGs in hippocampus and meninges of AD transgenic mice compared to WT controls at 5 and 8months versus 2 months baseline. Red dots represent significantly upregulated genes, blue dots represent significantly downregulated genes, and gray dots represent non-significant genes. **C** Line plots showing the number of differentially expressed genes at different age. **D** Expression patterns of disease-specific genes across different samples. **E** Correlation of logFC values between datasets at different time points. **F** Venn diagram showing the intersect of disease-specific up-regulated genes in hippocampus. Correlation analysis of log2 fold change values between datasets. **G** Functional enrichment analysis of the 19 validated genes using Metascape platform. FC: fold change

To validate these findings, we analyzed an independent bulk RNA-seq dataset (dataset2: GSE198226) containing hippocampus samples from AD and WT mice at 2-, 5-, and 8-months. Gene expression patterns correlated strongly between dataset1 and dataset2 in both WT and AD samples (WT: *R* = 0.69, *P* < 2.2 × 10^− 16^; AD: *R* = 0.69, *P* < 2.2 × 10^− 16^), supporting reproducibility across different experimental settings (Fig. 2A). Differential expression analysis in the validation cohort showed progressive increases in DEG numbers from 5 M (WT: 15; AD: 26) to 8 M (WT: 12; AD: 408), mirroring the pattern observed in the discovery dataset (Fig. 2B, C). Disease-specific genes identified in the discovery cohort showed consistent expression patterns in the validation dataset, with low expression in 2 M and WT samples, and elevated expression in late-stage AD samples (5 M and 8 M AD) (Fig. 2D). Correlation analysis of log_2_FC values between datasets showed weak positive correlations at early time points (*R* = 0.16, *P* = 0.012 at 2 M/3 M) but strong positive correlations at later time points (*R* = 0.55, *P* < 2.2 × 10^− 16^ at 5 M/6 M; *R* = 0.71, *P* < 2.2 × 10^− 16^ at 8 M/9 M), indicating that disease-associated gene expression changes become more consistent as pathology progresses (Fig. 2E). Taking the intersection of disease-specific genes from both datasets, we identified 19 robustly upregulated genes and no consistently downregulated genes, and cross-dataset correlation analysis revealed strong concordance in gene expression patterns of 19 genes (*R* = 0.76, *P* < 2.2 × 10^− 16^; Fig. 2F; Fig. S1). Gene ontology enrichment analysis revealed that these 19 genes are significantly enriched for immune response, inflammatory response, and phagocytosis, suggesting a homeostatic feedback mechanism to constrain excessive inflammation in AD (Fig. 2G).

To test whether the disease-specific transcriptional remodeling and core upregulated signature described above were robust across AD-related model, we introduced the independent tau-pathology dataset GSE218728. At the common-gene level, GSE218728 showed high overall expression concordance with the discovery dataset in both WT samples (*R* = 0.77, *P* < 2.2 × 10^− 16^) and disease samples (*R* = 0.76, *P* < 2.2 × 10^− 16^; Fig. S2A), with both correlations reaching high statistical significance. Differential expression changes were limited between 4-month PS19 and WT mice, but expanded markedly at 9-month, with significant upregulation of glial activation, lysosomal, and complement-related genes including *Gfap*, *Laptm5*, *Hexb*, and *C1qa*. Across age within PS19 mice, 9-month animals showed 1,066 DEGs relative to 4-month animals, including 972 upregulated and 94 downregulated genes; by contrast, WT aging detected only 10 DEGs (Fig. S2B). These results indicate that the major transcriptional perturbation in GSE218728 was linked to disease progression rather than normal aging. Importantly, hippocampal disease-specific upregulated genes defined in the discovery dataset were also elevated in late-stage PS19 samples and separated 9-month PS19 mice from WT and early-stage PS19 mice (Fig. S2C). Log_2_FC values between the discovery 9-month AD vs. WT contrast and the GSE218728 9-month PS19 vs. WT contrast were positively correlated for disease-specific genes (*R* = 0.67, *P* < 2.2 × 10^− 16^), with an even stronger correlation for the core 19-gene signature (*R* = 0.73, *P* = 1.6 × 10^− 6^; Fig. S2D). Thus, the disease-specific immune/glial response and the 19-gene core signature were reproduced not only in 5xFAD-related datasets but also in a PS19 tau-pathology model, supporting a relatively conserved molecular response associated with AD-related neurodegenerative progression.

### Proteomics supports RNA-level changes at protein level

**Fig. 3:**
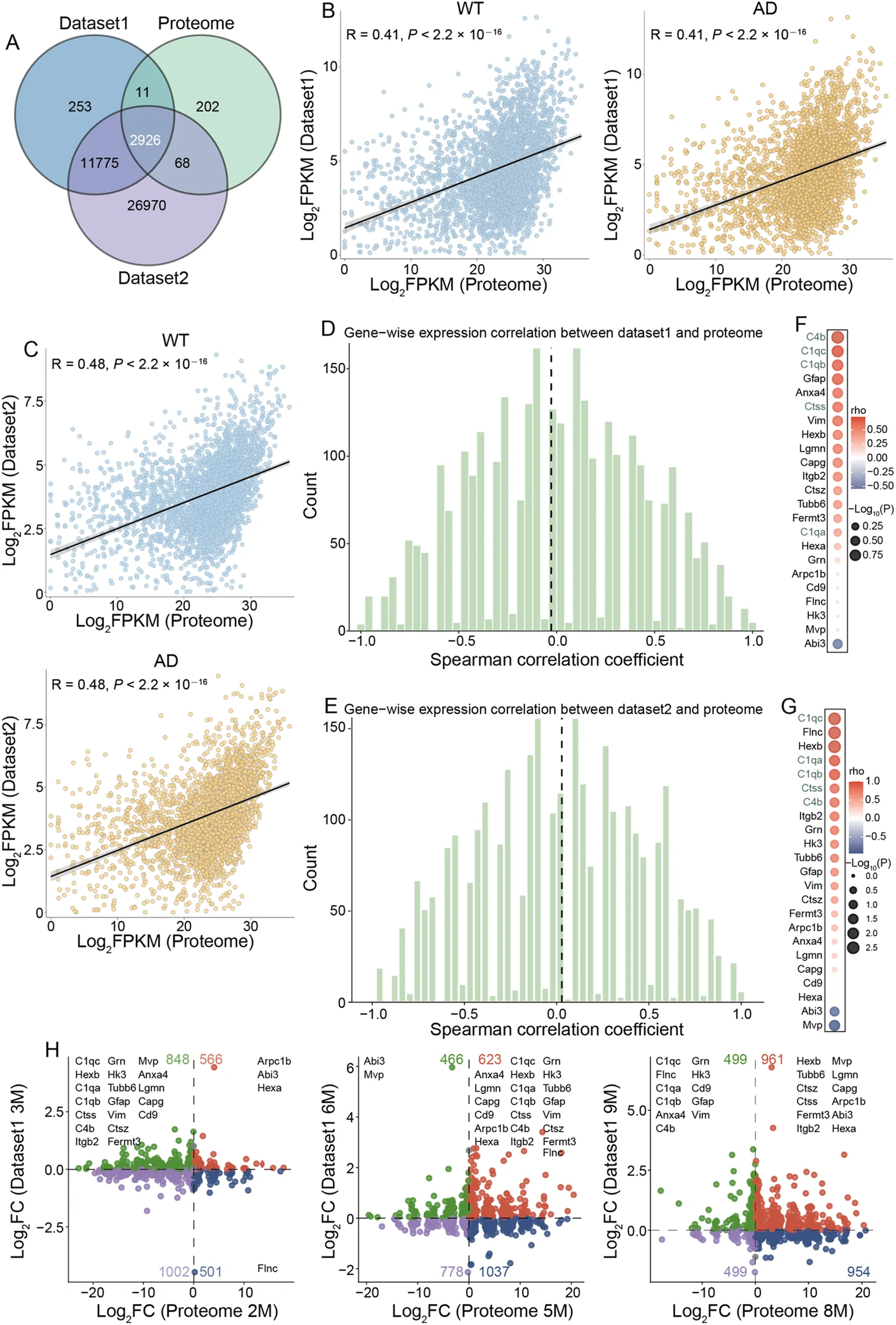
Proteomics validation of RNA-level findings. **A** Venn diagram showing gene overlap across three datasets. Only genes with detectable expression/protein levels in all datasets were included in correlation analyses. **B**,** C** Scatter plots showing correlation of gene expression between (**B**) bulk RNA-seq dataset 1 and proteomics dataset, (**C**) bulk RNA-seq dataset 2 and proteomics dataset. Each point represents one gene with detectable RNA and protein levels. **D**,** E** Distribution histograms of RNA-protein correlation coefficients for (**D**) dataset 1 vs. proteomics and (**E**) dataset 2 vs. proteomics. Vertical dashed lines indicate median correlation values. **F**,** G** Spearman correlations of disease-specific genes between RNA and protein levels for (**F**) dataset 1 vs. proteomics and (**G**) dataset 2 vs. proteomics. Green labels indicate genes from the 19 validated genes detected in proteomics. **H** Scatter plots showing the expression patterns in bulk RNA-seq dataset1 and proteomics dataset different time points

To assess whether transcriptomic changes translate to protein-level alterations, we collected hippocampal proteomics data from 5xFAD and WT mice at 2-, 5-, and 8-months using LFQ. After filtering for proteins with more than 2 unique peptides and present in > 50% of samples, 3,207 proteins with corresponding RNA measurements were retained for integrative analysis. We examined the overlap of expressed genes across the three datasets (dataset1 RNA-seq, dataset2 RNA-seq, and proteomics) and identified a common set of genes (2,926 genes) for cross-validation (Fig. 3A). Correlation analysis between bulk RNA-seq and proteomics datasets demonstrated good concordance in gene expression in both WT and AD samples (dataset1 RNA-seq: *R* = 0.41, *P* < 2.2 × 10^− 16^; dataset2 RNA-seq: *R* = 0.48, *P* < 2.2 × 10^− 16^), supporting the validity of our RNA-level findings (Fig. 3B, C). The gene-wise correlation analysis between RNA (FPKM) and protein (LFQ intensity) expression across samples revealed the symmetry of positive and negative correlation, and the median value of correlation coefficient is around 0 (Fig. 3D, E). Most hippocampus disease-specific upregulated genes showed positive RNA-protein correlations in both datasets (Fig. 3F, G). Among the 19 validated genes, five were detected in the proteomics data (highlighted in green), all showing consistent positive correlations. Classification of genes based on RNA and protein fold change directions identified five classes: Class I (RNA and protein concordant up), Class II (RNA and protein concordant down), Class III (only RNA up), Class IV (only protein up), and Class V (no significant change). In dataset1, disease-specific genes in Class I were mainly concentrated in microglia, complement function and lysosomes. Similar patterns were observed in dataset2, with the number of proteins located in the consistent up-regulated region and its up-regulated amplitude increased significantly (Fig. 3H; Fig. S3). This multi-level validation strengthens confidence in the identified disease signature and suggests that these genes represent robust molecular markers of AD pathology.

### Spatial transcriptomics maps hippocampus-meninges communication

**Fig. 4:**
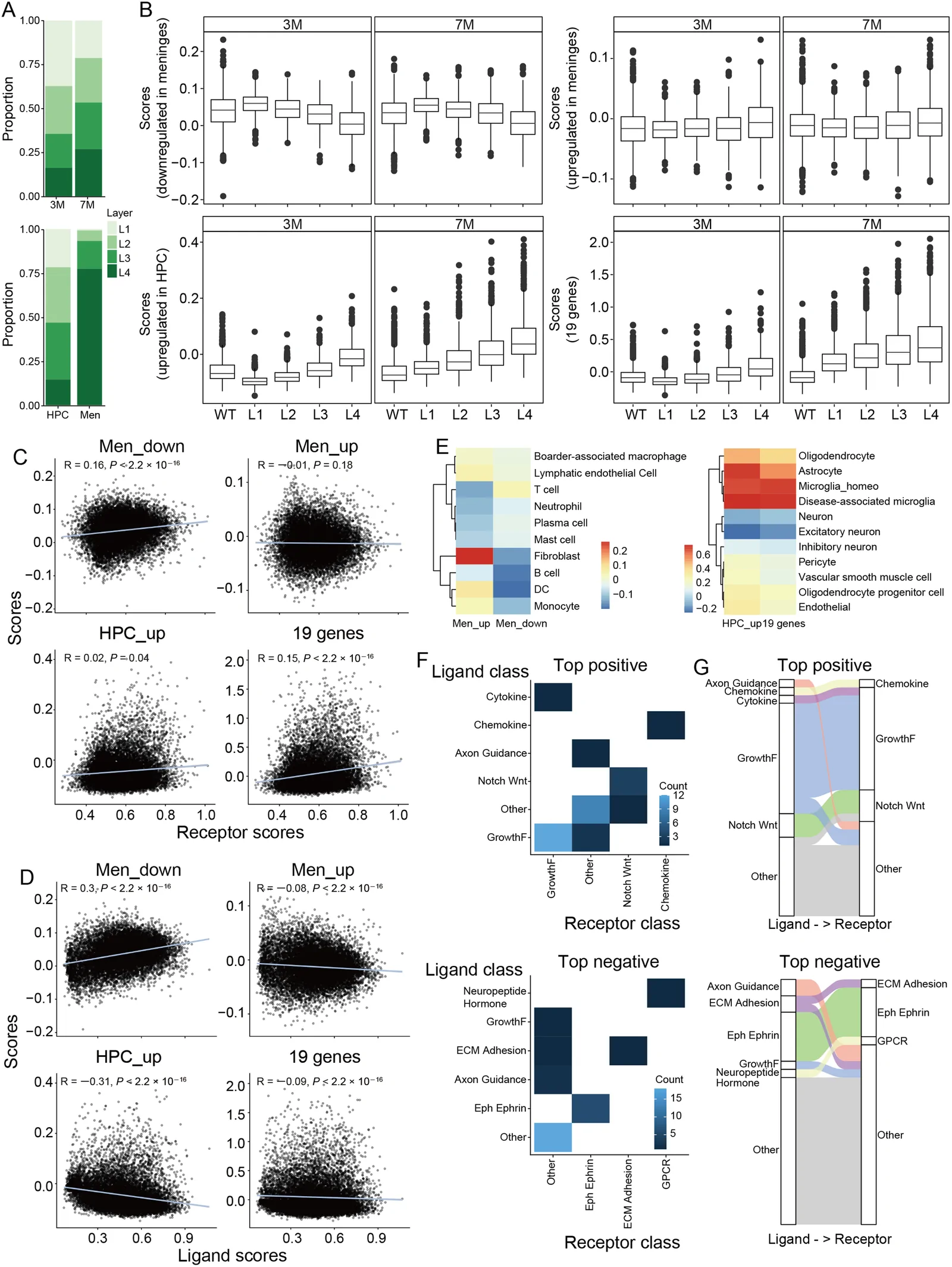
Spatial transcriptomics reveals hippocampus-meninges communication.** A** Stacked bar chart showing distribution of disease progression stages (L1-L4) across different time points (3, 7 months) and anatomical brain structures (hippocampus, meninges). **B** Box plots displaying disease-specific gene module scores across disease progression stages. **C**,** D** Scatter plots showing Spearman correlations between (**C**) receptor expression scores and (**D**) ligand expression scores with disease-specific gene modules. **E** Correlation matrix showing associations between major brain cell type signatures and disease-specific gene expression. **F**,** G** Heatmap and sankey diagrams displaying top the class of ligand-receptor pairs significantly correlated with hippocampus disease-specific genes

To understand the spatial organization of disease-specific genes, we analyzed spatial transcriptomics data (10x Visium) from coronal brain sections of 5xFAD and WT mice at 3- and 7-months. After preprocessing, batch correction, and clustering, we annotated anatomical regions using established marker genes, identifying hippocampus (cluster 3) and meninges (cluster 8) (Fig. S4). We developed a DPA metric defined as the difference between normalized immune score and normalized synapse score to quantify disease severity across spatial locations. Using this metric, we stratified samples into four disease stages (L1-L4) based on quartiles within each time point (3 M and 7 M AD). Analysis of stage distributions revealed that 7 M AD samples had a higher proportion of L3-L4 stages compared to 3 M AD, confirming disease progression over time (Fig. 4A). Different anatomical structures showed distinct layer composition, with clear enrichment of advanced layers in meninges. We examined the expression of disease-specific gene modules across DPA stages by computing module scores for hippocampal up-regulated (HPC_up), meningeal up-regulated (Men_up), meningeal down-regulated (Men_down), and the core 19 genes signature. Notably, HPC_up and 19 genes showed steeper increases in all or hippocampus spots, while Men_up and Men_down showed complementary patterns in all or meninges spots (Fig. 4B; Fig. S5A, B). This suggests coordinated molecular changes across brain-meningeal interface during disease progression.

To test whether the spatial disease progression axis and disease modules were transferable to an independent spatial transcriptomics dataset, we used GSE218360 and projected its spot expression profiles onto the DPA reference stratification constructed from 7-month GSE174321 samples. Projection scoring of the disease modules showed the same directional gradients: HPC_up, Men_up, and core 19-gene scores increased from L1 to L4 across spots, whereas Men_down decreased (Fig. S5C). Region-stratified analysis further showed that HPC_up and core 19-gene scores increased with DPA level in hippocampal regions, whereas Men_up was elevated in L3/L4 within meningeal regions (Fig. S5D). These findings demonstrate that the disease modules are not confined to the discovery dataset, but can reproduce pathology-gradient-related expression patterns in an independent spatial context.

The spatial proximity of meninges to hippocampus and the enrichment of immune cells in meningeal structures led us to investigate whether ligand-receptor signaling is associated with interregion communication. We compiled 846 valid ligand-receptor pairs from the CellPhoneDB curated database, requiring both ligand and receptor to be expressed in our spatial transcriptomics data. Correlation analysis revealed that hippocampus disease gene modules correlated more strongly with receptor scores, whereas meningeal disease modules correlated with ligand scores, suggesting receptor-program enrichment in hippocampal spots and ligand-program enrichment in meningeal spots in AD (Fig. 4C, D). This pattern persisted after stratified analysis to control for confounding in both two spatial transcriptomic datasets (Fig. S6-9). These validation results support a model in which meningeal ligand programs and hippocampal/disease receptor programs change coordinately in an independent spatial dataset. Cell type enrichment analysis revealed that 19 genes and HPC_up modules correlated most strongly with M0, DAM, astrocytes, and oligodendrocytes, while T cell, boarder-associated macrophage (BAM), DC, monocyte and fibroblast showed opposite patterns with Men_up and Men_down in meninges (Fig. 4E). This suggests that the hippocampus upregulated genes are primarily associated with multiple glial cell types in AD (Fig. 4E). To identify communication networks linking meningeal signals to hippocampal pathology, we computed LR activity scores. Correlation analysis between LR activity and disease modules (HPC_up, 19 genes) identified top 30 positive and negative associations. Top positive LRs were mainly enriched in the growth factors category, while the neg were mainly enriched in the Eph/Ephrin family (Fig. 4F, G; Fig. S10). These findings reveal asymmetric ligand-receptor expression patterns between meninges and hippocampus, supporting a candidate model of directional meninges-to-hippocampus communication associated with AD pathology.

### Microglial states show distinct TF-LR regulatory networks

**Fig. 5:**
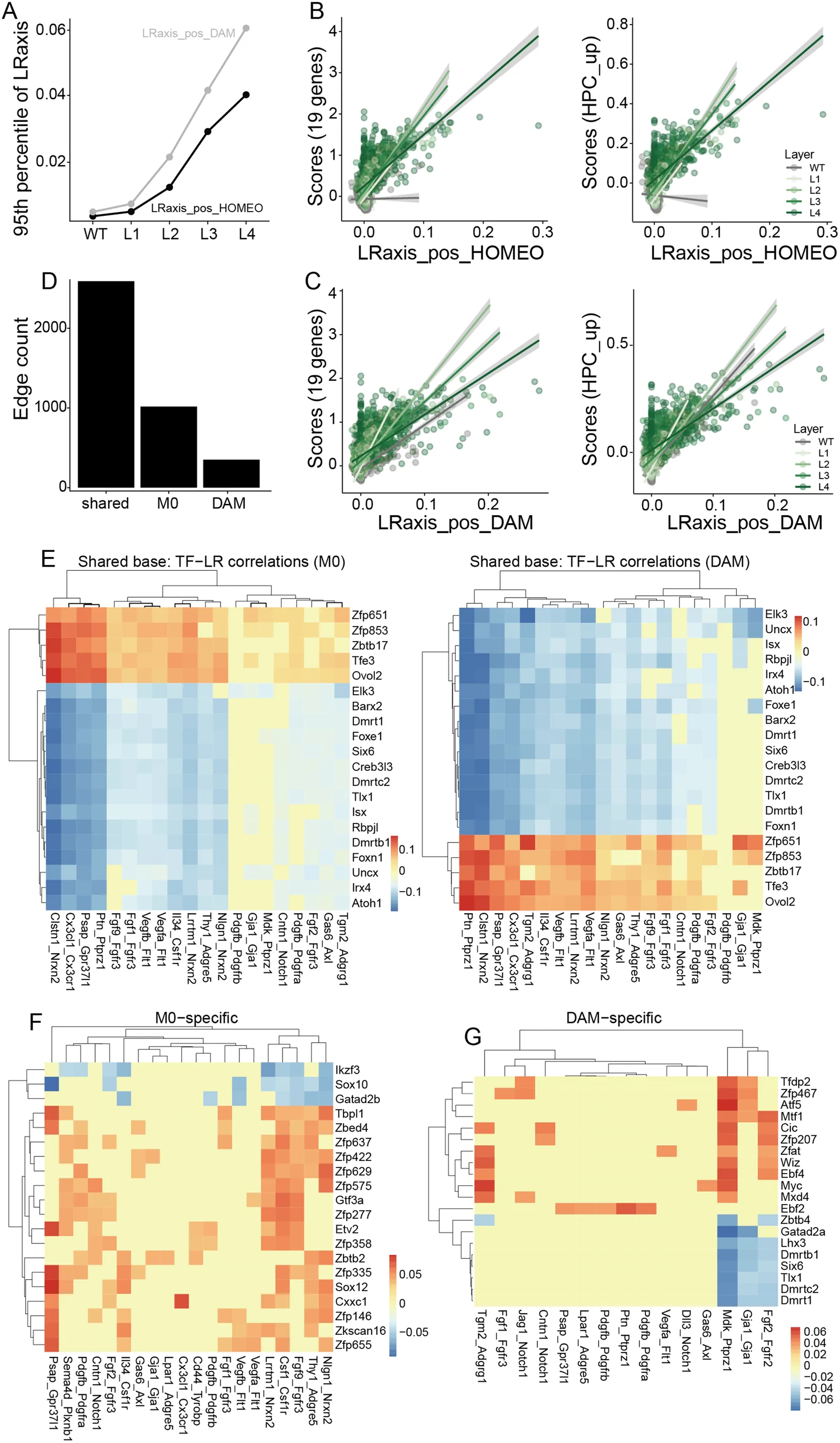
Transcription factor-ligand-receptor networks in microglia states.** A** Line plot showing 95th percentile of LRaxis scores (ligand-receptor interaction strength) across disease progression stages (L1-L4). **B**,** C** Stratified correlation analysis between LRaxis scores and disease-specific gene programs in (**B**) M0 microglia and (**C**) DAM microglia states. **D** The number of shared and state-specific TF-LR connections in M0 and DAM microglia. **E** Heatmap displaying shared TF-LR correlation patterns across M0 and DAM microglia states. Rows represent TF, columns represent LR pairs. **F** Heatmap displaying M0-specific TF-LR correlation patterns. Rows represent TF, columns represent LR pairs. **G** Heatmap displaying DAM-specific TF-LR correlation patterns. Rows represent TF, columns represent LR pairs

We focused on microglial regulatory networks given their strong correlation with disease signatures. We examined the top 30 positively correlated LR pairs and their associations with M0 and DAM microglia. LRaxis scores at the 95th percentile increased progressively from WT through L1-L4 for both M0 and DAM-associated LR axes, indicating that disease-associated communication signals concentrate in spatial hotspots that intensify with pathology (Fig. 5A; Fig. S11A). Stratified correlation analysis showed positive relationships between LRaxis and disease programs (19 genes and HPC_up), with significantly steeper slopes at L3/L4 compared to L1/L2, suggesting amplification of communication signals during disease progression (Fig. 5B, C). We inferred transcription factor activities and identified TF-LR connections specific to each microglial state. We classified connections as shared (present in both M0 and DAM), M0-specific, or DAM-specific (Fig. 5D). Shared connections were most abundant, followed by M0-specific and then DAM-specific connections, suggesting a core regulatory network operating across states with state-specific modifications. Heatmap analysis of shared TF-LR connections revealed similar block-like correlation structures in both M0 and DAM states, suggesting that shared connections represent a foundational upstream input layer functioning regardless of activation state (Fig. 5E). For the representative shared edge *Ptn*-*Ptprz1*, we confirmed coupling by computing residual correlations after controlling for layer and cell composition. Residual LR activity was associated with residual TF activity across all layers, demonstrating that this coupling is independent of disease stage (Fig. S11B). After controlling the layers, the effects of the M0-weighted axis and the DAM-weighted axis on the program become significant (Fig. S11C). This supports the interpretation that microglial-state-related communication axes explain variation in core disease programs beyond these covariates. M0-specific TF-LR networks showed more distributed, modular connectivity patterns, consistent with a homeostatic regulatory network (Fig. 5F). The *Csf1*-*Csf1r* signaling, as a representative M0-specific edge, is critical for microglial survival and proliferation (Fig. S11D) [17]. DAM-specific TF-LR networks exhibited more concentrated, hub-dominated connectivity, suggesting a reprogramming event associated with signaling through fewer key regulators during disease-associated activation (Fig. 5G). Representative DAM-specific edges showed weaker coupling compared to M0-specific connections (Fig. S11E). These findings support a two-layer regulatory mechanism: a shared upstream input substrate delivers baseline signals to microglia regardless of activation state, while state-specific downstream transcription factor reconfiguration determines the functional outcome—homeostatic maintenance in M0 or disease-associated activation in DAM.

## Discussion

This study presents a comprehensive characterization of AD progression in 5xFAD mice, integrating bulk transcriptomics, spatial transcriptomics, and proteomics with external validation analyses to identify disease-specific molecular programs and their spatial organization. Our findings indicate that disease-specific transcriptional changes are fundamentally distinct from normal aging, highlight the meninges as an immune-active interface associated with AD pathology, and suggest ligand-receptor signaling as a candidate mechanism of meninges-hippocampus crosstalk.

The identification of 251 hippocampal and 189 meningeal disease-specific genes provides a molecular framework for distinguishing AD pathology from normal brain aging. While aging-associated transcriptional changes occur in both WT and AD brains, disease-specific changes are restricted to AD and include genes involved in immune activation, complement cascade, and phagocytosis. The core 19-gene signature includes multiple functional categories that reflect key pathological processes in AD. Complement components (*C1qa/b/c*) and DAM markers (*Trem2*, *Tyrobp*, *Clec7a*, *Cst7*) confirm microglial activation as a central feature [18–20]. Lysosomal enzymes (*Ctss*, *Ctsb*, *Ctsd*, *Ctsz*) suggest enhanced phagocytic and degradative capacity [21]. Notably, the presence of resolution mediators (*Nfkbia*, *Anxa1*) indicates activation of anti-inflammatory pathways, potentially representing endogenous attempts to resolve inflammation. *Nfkbia* as an inhibitor of NF-κB signaling, its upregulation may represent a negative feedback mechanism to limit inflammatory responses [22–24]. However, chronic upregulation could also reflect sustained inflammatory stress. Similarly, *Anxa1* is a well-established pro-resolution mediator that promotes inflammation resolution and tissue repair [25–27]. The early upregulation of these genes suggests that resolution pathways are activated in early disease stages, though they may be insufficient to prevent disease progression. Proteomics validation suggested that transcriptional changes translate to protein level, with moderate but significant mRNA-protein correlations. This correlation is consistent with expected post-transcriptional regulation and confirms that the 19 genes signature is linked to functional protein-level changes rather than transcriptional noise.

*C1q* initiates the classical complement pathway and has been implicated in synapse elimination during development and in AD [28–30]. Our spatial analysis reveals that complement gene expression increases along the disease progression axis and correlates with meninges-hippocampus LR signaling, suggesting that complement-mediated synaptic pruning may be associated with signals from meningeal immune populations. This finding extends previous work on complement in AD by providing spatial context and identifying potential cellular sources.

The spatial transcriptomics analysis supports a view of meninges as immune-active structures associated with AD pathology. Meningeal regions expressed ligand programs linked to receptor programs in adjacent hippocampal tissue. The *Csf1*-*Csf1r* axis, identified as a top LR pair, is relevant because *Csf1r* inhibitors have been reported to modulate microglial activation in AD models [17, 31, 32]. These findings highlight meninges-derived signals as hypothesis-generating candidate modulatory pathways. The *Ptn*-*Ptprz1* axis is also relevant because it has been implicated in demyelination and neuroinflammation, and our data suggest this pathway is associated with meningeal-hippocampal communication [33, 34]. The *Ptn*/*Mdk*-*Ptprz1* axes may therefore represent candidate signaling interfaces for follow-up studies, but functional perturbation is required before assigning causal or translational roles.

Several limitations should be noted. First, our study used a mouse AD model, and findings should be validated in human tissue. Second, spatial transcriptomics has limited cellular resolution, and single-cell or spatial proteomic methods would provide finer detail. Third, our LR analysis is observational and correlational: it infers communication from expression patterns and does not directly measure protein interactions, ligand-receptor binding, or functional effects. Candidate signaling axes therefore require further functional experiments, such as ligand/receptor perturbation, spatial protein validation, or animal intervention studies, before causal or interventional roles can be assigned.

In conclusion, this study supports the meninges as a candidate modulatory interface associated with hippocampal pathology in AD and identifies spatially-organized molecular programs linked to disease progression. The disease-specific gene signature and LR networks identified here provide candidate signaling axes for biomarker development and functional validation. Future work should validate these findings in human AD samples and test the functional relevance of meninges-brain crosstalk in disease-relevant phenotypes.

## Supplementary Information

Below is the link to the electronic supplementary material.

Supplementary Material 1

Supplementary Material 2

## Data Availability

All datasets used in this study are publicly available. E-MTAB-16006 from ArrayExpress (https://www.ebi.ac.uk/biostudies/arrayexpress), GSE198226, GSE218728, GSE218360 and GSE174321 from Gene Expression Omnibus (https://www.ncbi.nlm.nih.gov/geo/), and PXD030348 from ProteomeXchange (https://www.proteomexchange.org/). Relevant codes used for data analysis are available from https://github.com/yccbio/AD/blob/main/multiomics_code.
