# Supplementary material for: Integrative transcriptomic analysis identifies meningeal-hippocampal immune communication in Alzheimer’s disease
Source: J Transl Med. 2026 Jun 25;24:1172. doi: 10.1186/s12967-026-08414-5 (PMC13563725; doi:10.1186/s12967-026-08414-5)
Supplement: Supplementary file 1 [file 12967_2026_8414_MOESM2_ESM.docx]

**Fig. S1** Venn diagram showing the intersect of disease-specific downregulated genes in hippocampus.

**Fig. S2** **Tau-pathology PS19 mouse cohort validates the disease upregulated genes identified in 5xFAD mice.** **A** Scatter plot showing the Spearman correlation of gene expression profiles between the 5xFAD discovery dataset and the independent PS19 tau-pathology validation dataset. **B** Bar plots showing the number of differentially expressed genes. **C** Expression patterns of 5xFAD-derived disease-specific genes across PS19 samples. **D** Correlation analysis of log2 fold-change values between the 5xFAD and PS19 datasets.

**Fig. S3** Scatter plots showing the expression patterns in bulk RNA-seq dataset2 and proteomics dataset different time points.

**Fig. S4 Spatial transcriptomics preprocessing and annotation. A** Batch correction analysis showing successful integration of spatial transcriptomics samples across different processing batches. **B** Unsupervised clustering analysis with spatial distribution overlay. **C** Heatmap showing expression of canonical marker genes across identified clusters.

**Fig. S5 Disease-module gradients along the spatial disease progression axis and validation in an independent spatial transcriptomics dataset. A** Spot-level module scores of hippocampal disease modules across DPA layers in the GSE174321 spatial transcriptomics dataset. **B, D** Region-stratified analysis of disease-module scores in hippocampal and meningeal spots from (B) GSE174321 and (D) GSE218360. **C** Projection-based validation of disease-module gradients in the independent GSE218360 spatial transcriptomics dataset. Spot-level expression profiles from GSE218360 were projected onto the DPA reference stratification constructed from 7-month GSE174321 samples.

**Fig. S6** Scatter plots showing Spearman correlations between ligand expression scores with disease-specific gene modules in GSE174321 dataset.

**Fig. S7** Scatter plots showing Spearman correlations between receptor expression scores with disease-specific gene modules in GSE174321 dataset.

**Fig. S8** Scatter plots showing Spearman correlations between ligand expression scores with disease-specific gene modules in GSE218360 dataset.

**Fig. S9** Scatter plots showing Spearman correlations between receptor expression scores with disease-specific gene modules in GSE218360 dataset.

**Fig. S10** Heatmap and Sankey diagrams displaying top the class of ligand-receptor pairs significantly correlated with hippocampus 19 genes.

**Fig. S11 Additional TF-LR network analyses. A** Line plot showing 95th percentile of LRaxis scores (ligand-receptor interaction strength) across disease progression stages (L1-L4) in validation dataset. **B, D, E** Representative scatter plots showing transcription factor-ligand-receptor residual correlations after controlling for disease stage and spatial effects. **C** Layer-controlled effects analysis demonstrating that TF-LR correlations remain significant after accounting for cortical layer identity. The top panel of the diagram shows the results of the discovery set, and the bottom panel shows the results of the validation set.
